# Current state of neonatal intestinal rehabilitation care in North America: A descriptive survey‐based study

**DOI:** 10.1002/ncp.70061

**Published:** 2025-10-28

**Authors:** Katie A. Huff, Ethan A. Mezoff, Abeer Azzuqa, Darren Bodkin, Hannah Hightower, Mark R. Corkins, Jeffery A. Rudolph, Katie M. Strobel, Jae H. Kim, Muralidhar H. Premkumar, Sujir Pritha Nayak, the Children's Hospitals Neonatal Consortium Intestinal Failure Focus Group

**Affiliations:** ^1^ Division of Neonatal‐Perinatal Medicine Indiana University School of Medicine Indianapolis Indiana USA; ^2^ Department of Pediatrics University of Cincinnati College of Medicine, Division of Neonatology, Perinatal Institute, Cincinnati Children's Hospital Medical Center Cincinnati Ohio USA; ^3^ Nationwide Children's Hospital, Division of Gastroenterology, Hepatology, and Nutrition Division of Clinical Informatics Columbus Ohio USA; ^4^ Department of Pediatrics University of Pittsburgh School of Medicine & UPMC Children's Hospital of Pittsburgh, Division of Newborn Medicine Pittsburgh Pennsylvania USA; ^5^ Department of Pediatrics Section of Neonatal Perinatal Medicine, University of Oklahoma Health College of Medicine Oklahoma City Oklahoma USA; ^6^ Division of Neonatology, Children's of Alabama Birmingham Alabama USA; ^7^ University of Tennessee Health Science Center, Division of Gastroenterology Memphis Tennessee USA; ^8^ Department of Pediatrics, Division of Gastroenterology, Hepatology, and Nutrition University of Pittsburgh School of Medicine and UPMC Children's Hospital of Pittsburgh Pittsburgh Pennsylvania USA; ^9^ Division of Neonatology, University of Washington Seattle Washington USA; ^10^ Division of Neonatology, Baylor College of Medicine Texas Children's Hospital Houston Texas USA; ^11^ Department of Pediatrics University of Texas Southwestern Medical Center Dallas Texas USA

**Keywords:** infant, intestinal failure, newborn, rehabilitation

## Abstract

**Background:**

Multidisciplinary care under intestinal rehabilitation programs (IRPs) improves survival in pediatric intestinal failure (IF). Professional societies recommend the management of pediatric patients with IF by an IRP. Whether these recommendations are followed in cases of neonatal IF is currently unclear. The objective of this study was to describe the current state of neonatal IF care across North America through the Children's Hospitals Neonatal Consortium.

**Methods:**

A web‐based survey was sent in July 2023. This survey consisted of adaptive questioning and contained 10–54 questions. The survey covered topics regarding IF care: diagnosis, rehabilitation program management, protocol use, and follow‐up. To determine correlation of respondent role and answer given, Spearman correlation was used to analyze a portion of responses.

**Results:**

There was a response rate of 93% (42/45). A total of 79% (33/42) of centers report having an IRP caring for neonatal patients. The composition and care provided by the program varied by center, with 60% (25/42) having protocols for neonatal IF management. A total of 76% (32/42) of centers report a multidisciplinary intestinal rehabilitation follow‐up.

**Conclusions:**

Neonatal IF care varies across North America. Although the presence of a rehabilitation program is known to improve pediatric patient outcomes, a relevant minority of centers in this consortium do not have access to this care. Future studies comparing neonatal specific IF care strategies are critically important to optimize outcomes.

## INTRODUCTION

Pediatric intestinal failure (IF) occurs when intestinal function is reduced below that necessary for growth and hydration, resulting in dependence on supplemental parenteral support, including intravenous fluids and parenteral nutrition (PN).^1^ The survival of patients with IF has improved over the last two decades, from approximately 60% up to 75%–100%, dependent on the population studied.^2^, ^3^, ^4^, ^5^ Multidisciplinary intestinal rehabilitation programs (IRPs) have been a primary reason for this improved survival. Multiple centers have reported better outcomes since implementation of an IRP with an overall estimated 22% increase in survival.^6^, ^7^, ^8^, ^9^

Multidisciplinary care of pediatric patients with IF was first described more than two decades ago.^10^ An IRP is an interdisciplinary, collaborative patient care team that works to coordinate care for children with IF.^1^ The care provided includes not only nutrition support but also monitoring and support for associated acute and chronic morbidities, with the overall goal to improve patient outcomes and survival. Based on the improved outcomes provided by an IRP, multiple societies—including North American Society for Pediatric Gastroenterology, Hepatology, and Nutrition (NASPGHAN) and American Society for Parenteral and Enteral Nutrition (ASPEN)—have recommended the use of an IRP to care for pediatric patients with IF with an ongoing need for parenteral support.^11^, ^12^, ^13^ Even with these recommendations in place, the rate of use of IRP teams in the neonatal intensive care unit (NICU) remains unknown. This is an important population to consider because most IF is diagnosed during the neonatal period in the first month of life.^14^ In addition, a younger age at diagnosis is associated with increased risk for transplantation or death.^14^ The objective of this study was to describe neonatal intestinal rehabilitation care in level IV NICUs across North America. Our primary goal was to describe the presence and composition of IRP teams within the NICU. Our secondary goals were to define local practice variations in relation to care provided to neonatal patients with IF.

## METHODS

This is a cross‐sectional study using an electronic survey to perform a descriptive analysis of neonatal intestinal rehabilitation care in NICUs across Children's Hospitals Neonatal Consortium (CHNC) centers. At the time of this survey, the CHNC consisted of 45 level IV referral NICUs across all regions of the United States with a single center in Canada. A level IV NICU is defined based on the American Academy of Pediatrics (AAP) definition as a NICU that provides sustained life support with the capability to provide surgical repair of complex conditions and access to pediatric surgical subspecialists.^15^ Through center collaboration, the CHNC provides one of the largest frameworks for research and quality improvement for neonates with complex and rare medical diagnoses.^16^ For the purposes of this project, all centers were considered eligible. Since the survey was distributed, four additional centers have joined the CHNC but were excluded from the study because they joined after survey distribution.

The survey was primarily designed by two of the authors, with input regarding content from all authors. The survey was further reviewed by a larger group of professionals including neonatologists, gastroenterologists, surgeons, nurses, advanced practice providers (APPs), and other members who participate in the CHNC IF focus group. The survey was created and data were captured using REDCap electronic data capture tools hosted at Indiana University.^17^, ^18^ This study was reviewed by the Indiana University School of Medicine Institutional Review Board (IRB) and found to be exempt from further review (IRB #19382). Data were kept secure, with survey responses only available to a single author. Formal consent was not deemed necessary, as language in the survey noted its voluntary nature.

The survey consisted of a minimum of 10 questions divided into two sections and took 5–10 min to complete. Adaptive, branching questioning was used to limit the length of the survey and minimize the number of questions asked to each respondent. Based on respondent answers, the questionnaire had up to 54 questions. The instructions asked that an individual familiar with IF care in the NICU complete the survey and noted that more than one individual could complete the survey together. Respondents were instructed to answer all questions based on institutional practice and not individual preferences. The goal of the survey, outlined in the instructions, was to have a single survey completed for each CHNC institution.

The survey topics broadly included respondent role, local definition of IF, presence of an IRP team, and inpatient and outpatient care of neonatal patients with IF. The definition of an IRP team for the purposes of the survey was “a multidisciplinary team of at least two specialties who cares for patients with intestinal failure.” Inpatient care topics included the presence of a nutrition protocol for IF care in the NICU, care of patients with ostomies, and discharge planning, including PN teaching. Outpatient topics included clinic follow‐up and specialists available in clinic. A full copy of the survey is available in Supporting Information 1.

The survey was distributed to CHNC site sponsors via email in July 2023. Reminder emails were sent first en masse to all site sponsors after 2 weeks. Individual center emails were sent between July and September 2023 to centers that had not yet completed the survey. The center name was documented with each response to track centers that had responded. Duplicate responses from centers were compared. If duplicate answers did not agree, both answers to the question were excluded. If duplicate answers agreed, only one of the responses was included.

This study was meant to be a descriptive study of the current care provided to patients with IF in the NICU, and descriptive statistics were used to present most of the data. Spearman correlation was used to assess the correlation between respondent‐reported role and specific answers, including IF definition, IRP presence, IRP members, and clinic follow‐up. Respondent role was defined as the self‐identified clinical specialty reported as part of the survey. Data were analyzed using SPSS version 29.0.1.0.^19^ A *P* value of 0.05 was used to define statistical significance.

## RESULTS

A total of 43 responses were received from 42 centers, leading to a response rate of 93% (42/45 centers). Most respondents, 83% (35/42), were neonatologists (Table 1), and 54% (22/41) were members of the IRP team. In most centers, neonatology was the primary team caring for neonatal patients with IF, although multiple centers selected more than one care team option. Fifty‐four percent (22/41) of centers reported having no center‐specific definition for IF (Table 1). The respondent role identified did not correlate with IF definition (*P* = 0.058).

**Table 1:** Respondent and local center details (*n* = 42 unless otherwise specified). [Correction added on 14 November 2025, after first online publication: In “Survey Parameter” column the “F definition” was revised to “IF definition”.]

Survey parameter	Response	*n* (%)
Respondent role	Neonatologist	35 (83)
	Dietitian	6 (14)
	Gastroenterologist	5 (12)
	Surgeon	3 (7)
	APP	2 (5)
	Pharmacist	1 (2)
Primary care team for patients with IF in NICU	Neonatology	39 (93)
	Surgery	9 (21)
	Gastroenterology	6 (14)
IF definition (*n* = 41)	No definition defined	22 (54)
	PN 60 days in 74‐day period	9 (22)
	PN >60 days because of intestinal disease, dysfunction, or resection	7 (17)
	PN ≥90 days	3 (7)
	PN ≥42 days after bowel resection	0 (0)
	Center specific	0 (0)

Abbreviations: APP, advanced practice provider; IF, intestinal failure; NICU, neonatal intensive care unit; PN, parenteral nutrition.

Seventy‐nine percent (33/42) of centers reported an IRP team involved in NICU IF care. The composition and use of the IRP, however, varied by center and are described in Table 2. The respondent role reported did not correlate with reported IRP presence (*P* = 0.173) or specialists identified as part of the IRP (*P* = 0.568). Forty‐five percent of centers had criteria for consulting the IRP with the NICU most often the service to initiate consultation (91%). The most common timing for IRP consultation was the inability to advance feeds (50%) or after bowel resection or ostomy formation (44%). The IRP most often followed patients weekly at 75% (24/32) of centers.

**Table 2:** IRP description including use and team composition (total *n* = 33 for all parameters unless specified).

Survey parameter	Response	*n* (%)
IRP team members	Gastroenterology	33 (100)
	Dietitian	32 (97)
	Neonatology	29/32 (91)
	Surgery	27/32 (84)
	APP	24 (73)
	Pharmacy	22 (67)
	Nurse	15/32 (47)
	Other	2 (6)
	PT/OT	1 (3)
	Discharge manager	1 (3)
Years IRP team in practice (*n* = 32)	>10	14 (44)
	5–10	10 (31)
	2–5	6 (19)
	<2	2 (6)
Criteria to consult IRP	Yes	15 (45)
Alerts IRP to consult	NICU	30 (91)
	Dietitian	14 (42)
	Surgery	11 (33)
	Gastroenterology	2 (6)
Timing of initial IRP involvement	If unable to advance feeds	16/32 (50)
	At bowel resection or ostomy formation	14/32 (44)
	When intestinal failure definition is met	13 (39)
	When cholestasis develops	12 (36)
	When ready to transfer or discharge	7 (21)
	When starting feeds	2 (6)
	Other	3 (9)
	When additional services are believed to be needed	3 (1)
	As early as possible/from admission	2 (6)
Frequency of IRP follow‐up (*n* = 32)	Weekly	24 (75)
	As needed/dependent	11 (34)
	Close to discharge	3 (9)
	Other	5 (16)
	Daily	2 (6)
	Twice monthly	2 (6)
	Monthly	0 (0)

Abbreviations: APP, advanced practice provider; IRP, intestinal rehabilitation program; NICU, neonatal intensive care unit; PT/OT, physical therapy/occupational therapy.

**Figure 1:**
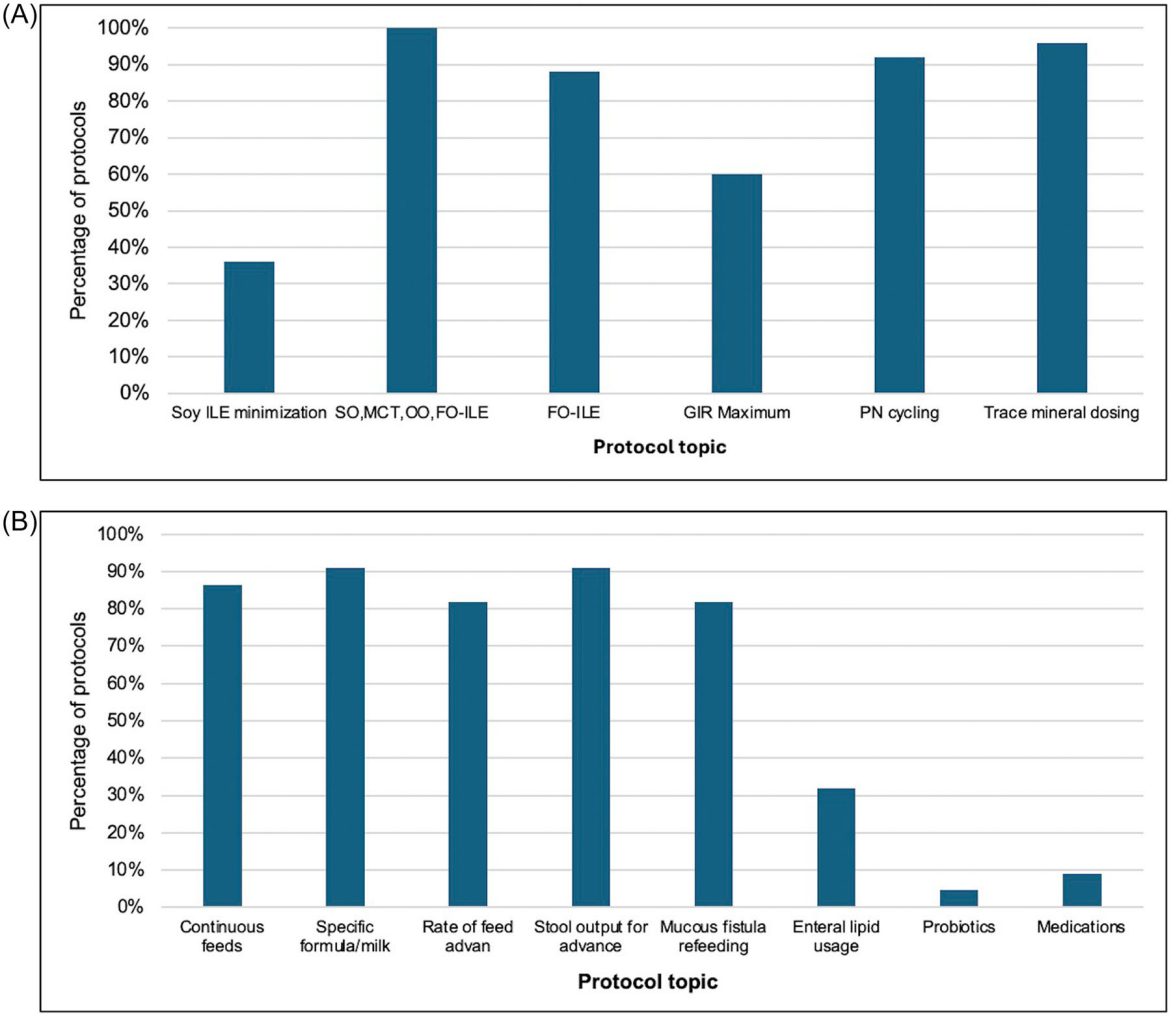
Nutrition protocol topics. (A) PN protocol topics (for each parameter *n* = 25). (B) Enteral nutrition protocol topics (for each parameter *n* = 22). FO‐ILE, fish oil–based intravenous lipid emulsion; GIR, glucose infusion rate; PN, parenteral nutrition; SO,MCT,OO,FO‐ILE, soy, medium‐chain triglyceride, olive, fish oil–based intravenous lipid emulsion.

Sixty percent (25/42) of centers had a nutrition or general protocol for caring for patients with IF in the NICU. However, the contents of this protocol vary by center, with 100% (25/25) of protocols including PN, intravenous lipid emulsion (ILE) usage, and laboratory monitoring; 88% (22/25) including enteral nutrition; 20% (5/25) including ethanol lock usage; and 8% (2/25) including heparin lock usage. The PN and ILE usage protocols varied by center, with soy, medium‐chain triglycerides, olive oil, fish oil–based ILE (SO,MCT,OO,FO‐ILE) being the only consistent surveyed care strategy at all centers (Figure 1A). For enteral nutrition protocols, no surveyed care strategies were included in all center protocols (Figure 1B). Of interest, 82% (18/22) of centers report including mucous fistula refeeding in their protocol.

Most centers (77%, 30/39) have a vascular access protocol for home, with surgeons being the primary team placing vascular access (81%, 34/42) and no specific preference for access (41%, 18/41) (Table 3). For home feedings, all centers offer gastrostomy tubes, but not all (88%, 37/42) offer oral feeding as a home option (Table 3). Most centers consider discharge or transfer from the NICU with a colostomy (90%, 38/42), but fewer consider with an enterostomy (64%, 27/42). Type of enterostomy further influenced offering transfer or discharge from the NICU (Table 3). Discharge teaching most often occurred in the NICU but varied by type of teaching, with ostomy teaching more likely in NICU (95%, 36/38) compared with PN teaching (60%, 25/42) (Table 3).

**Table 3:** Center‐specific practices for discharge planning of neonates with intestinal failure (for each parameter *n* = 42 unless specified).

Survey parameter	Response	*n* (%)
Vascular access		
Vascular access protocol for home	Yes	30/39 (77)
Places vascular access for home	Vascular team	6/41 (15)
	Surgeons	34 (81)
	Interventional radiology	17 (40)
Location preference for home vascular access (*n* = 41)	Any location/no preference	18 (41)
	Upper limb/tunneled line	14 (34)
	Internal jugular	13 (31)
	Lower limb	0 (0)
Home nutrition		
Location of home PN teaching	NICU	25 (60)
	General floor	20 (48)
	Rehabilitation facility	1 (2)
	Do not discharge home with PN	1 (2)
Feeding methods offered for home	Gastrostomy tube	42 (100)
	Oral feeding	37 (88)
	Continuous feeds	34 (81)
	Continuous via NG	11/33 (33)
	Gastrostomy‐jejunostomy tube	33/41 (80)
	NG tube	19/41 (46)
	Jejunal feeds (NJ/transpyloric)	17/41 (41)
Ostomy planning		
Discharge or transfer from NICU with ostomy	Yes: large intestine ostomy	38 (90)
	Yes: small intestine ostomy	27 (64)
	No	3 (7)
Discharge home by enterostomy type	Ileostomy	27/27 (100)
	Jejunostomy	14/27 (52)
	Duodenostomy	9/26 (35)
Transfer or discharge home with ostomy (*n* = 39)	Discharge home	39 (100)
	Transfer to floor/other unit	17 (44)
	Transfer to another institution	9 (23)
Location of ostomy training	NICU	36/38 (95)
	General/surgery floor	14/39 (36)
Clinic follow‐up		
Who follows if no IRP clinic (*n* = 10)	Gastroenterology	10 (100)
	Surgery	5 (50)
	Neonatology	1 (10)
	Complex care	1 (10)

Abbreviations: IRP, intestinal rehabilitation program; NG, nasogastric; NICU, neonatal intensive care unit; NJ, nasojejunal; PN, parenteral nutrition.

**Figure 2:**
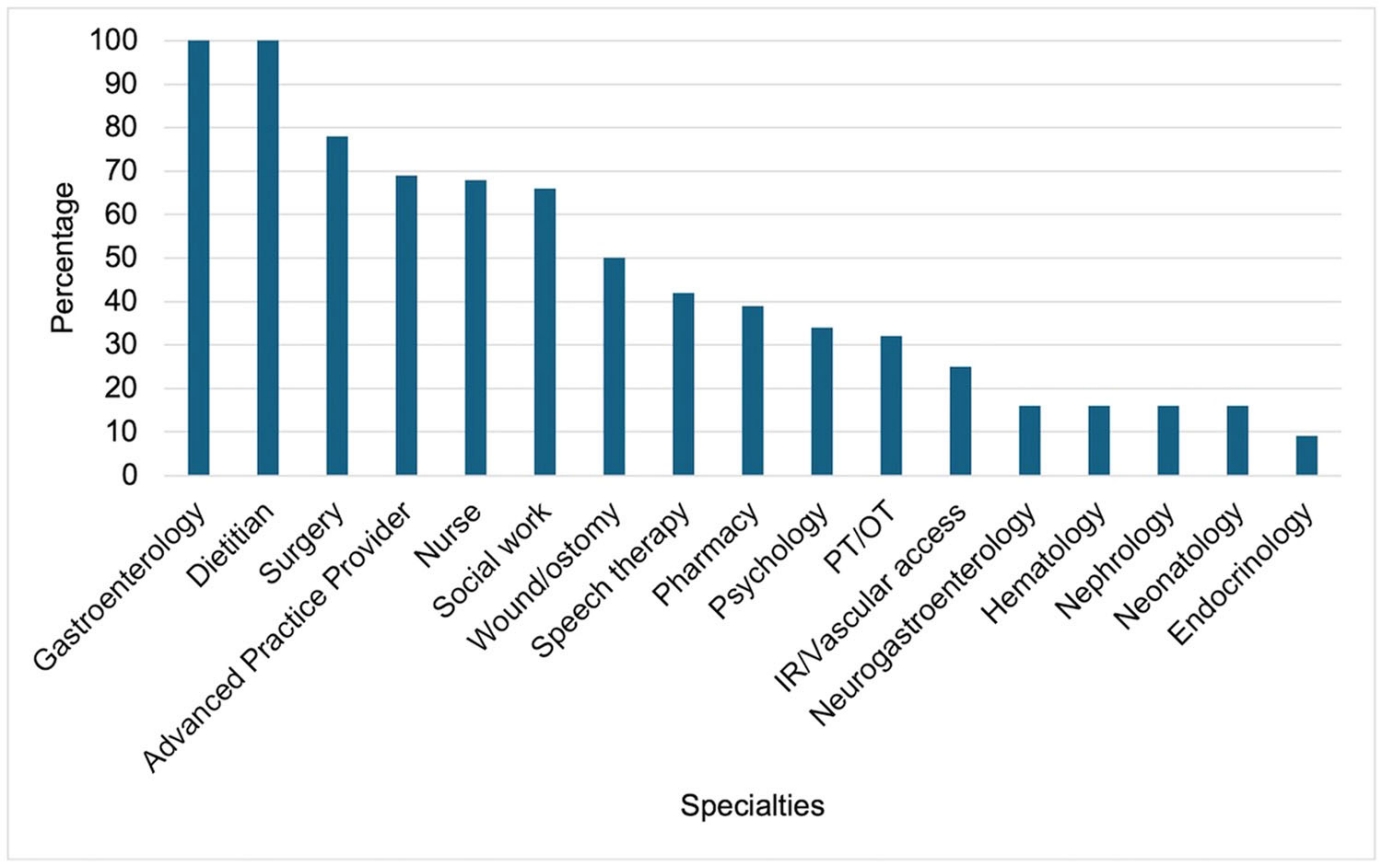
Specialists in intestinal rehabilitation program clinics. Presented as percentage of centers reporting presence of specialty (*n* = 32). IR, interventional radiology; OT, occupational therapy; PT, physical therapy.

Outpatient follow‐up also varied by center (Table 3). Of the 42 centers, 32 (76%) had an outpatient multidisciplinary clinic dedicated to patients with IF. The role of the survey respondent did significantly correlate with reporting the presence of an IRP clinic (*P* = 0.021). The specialties present in these IRP clinics varied by center (Figure 2), with all clinics including a gastroenterologist and dietitian. In those 10 centers that did not have a dedicated IRP clinic, gastroenterology most commonly provided the follow‐up (100%), followed by surgery (50%; Table 3).

## DISCUSSION

To our knowledge, this is the first report on the state of IF care within the NICU. Despite strong association with improved outcomes and multiple international societies recommending IF care by a multidisciplinary IRP team, including NASPGHAN and ASPEN, 21% of referral level IV NICUs in North America participating in CHNC do not have an IRP team collaborating in the NICU.^11^, ^12^, ^13^ Although some might question whether the clinical role of the survey respondent correlated with the knowledge needed to report the presence of an IRP team in the NICU, we found no correlation between respondent role and the report of an IRP team in the NICU.

Some of the heterogeneity in patient care observed in our survey cohort could be related to differences in definition of IF. Fifty‐four percent of centers reported having no standardized definition of IF in their NICU. The respondent role was not correlated with this response but was close to significance (*P* = 0.058). With many survey respondents being neonatologists, it could be they are not aware of the IF definition used by gastroenterologists and others on the IRP team. This is an important gap to consider, as a lack of understanding or ability to identify IF could delay consultation of the local IRP and alter the care provided to patients with IF in the NICU. Additionally, it is important to note that many patients who go on to meet the definition of IF require IRP consultation and management prior to meeting this threshold. To capture the use of IRP teams in this setting, we inquired about the timing of IRP consultation and the use of established criteria. Only 45% of centers had set criteria for IRP consultation, with criteria varying by center. This finding is concerning given a lack of consensus for local IRP consultation may further delay IRP involvement, regardless of whether a standard IF definition or the knowledge of this definition exists.

The configuration of the IRP varies based on local needs with each member playing an important and distinct role; most described in the literature include a pediatric surgeon, pediatric gastroenterologist, specialized nurses, and dietitians.^20^ These specialists are the minimum members NASPGHAN recommends including in an IRP.^11^ NASPGHAN also recommends close collaboration with neonatologists and note that the presence of additional specialists may be beneficial.^11^ In this survey, only 42% (14/33) of the IRP teams included all four of these recommended members. Interestingly, most IRPs in this survey included neonatologists (91%). Given our survey did not inquire about the reasoning for team member inclusion, we cannot directly report, and can only infer, why many IRP teams include a neonatologist. It is most likely the nature of the population (ie, NICU patients) or the respondents (83% neonatologists) or both that led to this high rate of reporting neonatologist involvement. Also, we cannot report or infer about the neonatologist's role in the IRP, as this information was also not included in our survey questions.

Prior groups have reported the nutrition practices used in caring for patients with IF.^21^ These surveys, however, focused on the nutrition management of pediatric patients at the reporting centers, whereas we report specifically on the neonatal population. Additionally, prior surveys asked about direct practice management strategies and not local protocols, limiting our ability to correlate the prior results to ours. The use of a postoperative nutrition protocol has been linked to improved outcomes in neonatal patients.^22^, ^23^ With a nutrition protocol in place, Shakeel et al were able to show a decrease in time to initiate feeds, time to advance feeds, time receiving PN, and the severity of IF‐associated liver disease.^22^ This is an important consideration as a possible means to improve patient outcomes, as only 60% (25/42) of centers reported a protocol for neonatal patients with IF in their center. The contents of these protocols varied between centers. Interestingly, most centers report including mucous fistula refeeding despite a lack of consensus on its usage. In a review, Ghattaura et al noted those who received mucous fistula refeeding had improved growth and shorter PN course; however, there were reports of major complications from a single study.^24^

A previous survey that included predominately CHNC centers reported ILE usage across the United States, with 79% (84/106) of centers having a written protocol for ILE usage.^25^ In comparison, in this survey, we found 60% (25/42) of centers report a nutrition protocol for patients with IF, with all (25/25) including ILE usage. Both surveys report a high rate of SO,MCT,OO,FO‐ILE usage in neonates with gastrointestinal disorders.^25^ In our current report, we are limited in the ability to directly compare ILE usage trends with those in our prior survey because we did not ask about clinical use strategies, only the presence of a protocol and strategies included. It does, however, seem there is a trend in increased availability, if not usage, of fish oil–based ILE (FO‐ILE) in the NICU, as 88% (22/25) of centers with PN protocols in our cohort compared with 55% (58/107) in the previous included FO‐ILE.

When planning discharge home, all centers (42/42) offer gastrostomy tube placement. However, only 88% (37/42) report offering oral feeding for home. The reason for this result is unclear and may be related to misunderstanding of the question and response options by individuals completing the survey. Infants with IF are well known to be at high risk for oral aversion, with recommendations made in the literature to offer oral feedings as early as possible to decrease the risk for aversion.^26^ This recommendation must be balanced with concerns of stool output stimulation with oral or gastric bolus feedings and attempting to improve intestinal adaptation with stimulation from early enteral feedings.^27^ Some centers may find oral feedings permissible, or they may offer them for some but not all patients. Furthermore, respondents may not have felt their center has structured support for home oral feedings in this population (eg, dedicated feeding clinic). Importantly, tight control of the feeding regimen, in the hospital and after discharge, is critical for patients with short bowel syndrome who are at risk of dehydration and harmful electrolyte deviations when feedings drive excessive malabsorptive stool output. Thus, the feeding regimen must strike the appropriate balance between more conservative strategies and strategies that risk malabsorption to mitigate the development of oral aversion and stimulate bowel adaptation. Interestingly, 81% (34/42) of centers offer continuous feeds for home, with 33% (11/33) of these centers offering continuous feeds via nasogastric tube for home. Although there is concern for the higher risk of nasogastric tube dislodgement, studies have shown increased risk of complications and return to care with gastrostomy tube usage in prior NICU patients.^28^ The existing literature suggests that the type of feeding tube is also not associated with a difference in outcomes, including the achievement of enteral autonomy or duration of PN in patients with IF.^29^

For further discharge planning, we also considered ostomy care. Most centers, 90% (38/42), consider discharge home from the NICU with a colostomy. This is in comparison with discharging home with enterostomies, which vary by type: 64% (27/42) discharging home with an ileostomy, 33% (14/42) with jejunostomy, and 21% (9/42) with duodenostomy. The reasoning for discharge home with a given ostomy type was not included in our survey. There are many individual patient factors that may influence the need for ongoing ostomy placement. These factors and individual patient needs, compared with commonplace institutional strategies and practices, may influence the responses received to this question. Additional detailed information, beyond that provided by our survey, about local practice strategies and stances would be helpful to fully draw conclusions from these reports of ostomy discharge rates. When PN and vascular access are needed upon discharge, most centers report a protocol for home access. However, many centers (41%) report no location preference for home vascular access. This contrasts with the NASPGHAN recommendations stating upper extremity access is the preferred location.^30^ Our survey question for this topic did not specify the location preference in the vascular access protocol. Despite our instructions to report institutional practice, it is possible individual preference or lack of presence may have been reported.

At the time of our survey, 76% of centers had a dedicated multidisciplinary IRP clinic that followed neonates on discharge from the NICU. This is in comparison with a survey conducted in 2015 that reported only 50% of centers surveyed had an organized IRP clinic for neonates.^21^ Since the time of this earlier survey, NASPGHAN published recommendations regarding IRP management of complex patients with IF, which could potentially explain this increase in organized IRP clinics over the last decade.^11^ The specialists included in these IRP clinics varied by center, which likely reflects the local preferences and needs of the providers and patients. Of note, all centers do include a gastroenterologist and dietitian as recommended by NASPGHAN; however, only a portion included pediatric surgery (78%) and nursing (68%), although 69% of centers reported including an APP (nurse practitioner or physician assistant).

This study does have limitations. Although we instructed the respondents to complete the survey based on their institutional practices, individual biases may have played a role in the responses given. To assist with limited individual knowledge of practice, including outpatient practice, we instructed respondents that more than one person could answer questions together. However, only five responses included more than one individual respondent. We did, however, attempt to control for these individual preferences and knowledge gaps by reporting correlation between respondent role and answers with most answers showing no correlation. Responses that either correlated with respondent role (eg, presence of an IRP clinic) or were close to correlation (eg, IF definition) likely reflect the limited knowledge of the neonatal professional with regards to the outpatient management of the patient with IF. This highlights the need to expand knowledge in the neonatal professional, as providing counseling on future expectations for families of IF neonates is important and necessary. A strength of our survey is the inclusion of a large number of centers across North America through the CHNC. These centers include level IV referral NICU centers, with surgical evaluation or management being the most common reason for referral, making this a reasonable cohort of centers for this survey.^16^ Variation in outcomes between CHNC centers has been noted in prior reports, and future attempts to link practice variation from this survey to patient outcomes are an important consideration.^16^, ^31^

## CONCLUSION

The care of neonates with IF is highly variable among centers across North America. Whereas most centers have a formal IRP that cares or consults for these patients in the NICU, a significant number of centers (21%) do not have an IRP in the NICU. In addition, considerable variation exists in the protocols and practices at the centers. Prior reports have correlated improved outcomes for patients with IF with an IRP presence. Further information is needed about best practices within these IRPs to optimize patient outcomes and recommendations for standardization of patient care. In particular, more information is needed regarding the ideal timing of IRP involvement in neonatal care and which specialists should be involved.

## AUTHOR CONTRIBUTIONS

Katie A. Huff and Sujir Pritha Nayak equally contributed to the conception and design of the research. Ethan A. Mezoff, Abeer Azzuqa, Darren Bodkin, Hannah Hightower, Mark R. Corkins, Jeffery A. Rudolph, Katie M. Strobel, Jae H. Kim, and Muralidhar H. Premkumar contributed to the design of the research. Katie A. Huff contributed to the acquisition and data analysis. All authors contributed to the interpretation of data. Katie A. Huff and Sujir Pritha Nayak drafted the manuscript. All authors critically revised the manuscript, agree to be fully accountable for integrity and accuracy of the work, and read and approved the final manuscript.

## CONFLICT OF INTEREST STATEMENT

Jae H. Kim is a medical advisor for Medela and Infant Biotherapeutics; consultant for Cardinal Health, Carag, and Biomilq; and shareholder for Astarte Medical and Nicolette.

## Supporting information

Supporting Information 1 IRP Survey.
