# Supplementary material for: Current state of neonatal intestinal rehabilitation care in North America: A descriptive survey‐based study
Source: Nutr Clin Pract. 2025 Oct 28;41(4):1180–90. doi: 10.1002/ncp.70061 (PMC13419105; doi:10.1002/ncp.70061)
Supplement: Supplementary file 1 — Supporting Information 1 IRP Survey. [file NCP-41-1180-s001.pdf]

CHNC Intestinal Failure NICU Care

Please ask an individual or group who would be familiar with the care of intestinal failure patients in the NICU at your CHNC institution to complete the following survey.

We ask for only one survey to be comleted per CHNC institution.

Please consider your CHNC institution and care provided in the NICU when answering the following questions. (Please do not answer based on your individual opinion).

The survey will take about 5-10 minutes to complete. There will be no personal benefit in participating in the study. However, it is anticipated this study will benefit research knowledge in general. This survey was approved by the IRB (# 19382).

The goal of this survey is to better understand intestinal failure management in the NICU across CHNC centers. Participation in the survey is voluntary. No personal information will be obtained in this survey. Survey responses will be kept in a secure manner via REDCap.

Thank you!

CHNC Hospital Name

To limit to only single center response, use name as listed on map here

If you receive a "duplicate" notification, a survey for your institution has already been completed, if this is felt to be an error please email

____
(if not listed on map please enter hospital name to best of knowledge)

If other, what is the name of your CHNC hospital?

____

What is your role within the NICU?

(For group check all positions for individuals completing)

- ☐ Neonatologist
- ☐ Surgeon
- ☐ Gastroenterologist
- ☐ Nurse
- ☐ Pharmacist
- ☐ Dietitian
- ☐ Advanced Practice Provider (NP/PA)
- ☐ Other

---

if other, what is your role?

____

Which definition does your NICU or institution use for intestinal failure?	◯ ASPEN: Need for TPN for 60 days within a 74 day period<br>◯ CHNC: Need for TPN for 90 days or more<br>◯ NASPGHAN: Need for PN >60 days due to intestinal disease, dysfunction, or resection<br>◯ Canadian Association of Surgeons: PN for 42 days after bowel resection<br>◯ Center Specific or Other (please add criteria below)<br>◯ We do not have a specific definition
If other or center specific, what definition does your unit use for intestinal failure?	__________________________________
Which clinical group serves as the primary care team in the NICU for patients with intestinal failure? (Check all that apply)	☐ NICU<br>☐ Surgery<br>☐ GI<br>☐ Other
If other, who is the primary care team for intestinal failure patients in the NICU?	__________________________________

For the purposes of the following section, an intestinal rehabilitation team refers to a multidisciplinary team (of at least 2 specialties) who cares for patients with intestinal failure.

Within your NICU, do you have a team composed of 2 or more specialties that cares for patients with intestinal failure?	◯ Yes<br>◯ No
What specialties constitute this "intestinal rehabilitation" team that cares for your NICU intestinal failure patients? (check all that apply)	☐ Gastroenterology<br>☐ Surgery<br>☐ Neonatology<br>☐ Dietician<br>☐ Pharmacy<br>☐ Advanced practice providers (NP/PA)<br>☐ Nurse<br>☐ Other
If other, what other specialties are part of your inpatient NICU intestinal rehabilitation team?	__________________________________
How many years has this multidisciplinary intestinal rehabilitation team been in practice within the NICU?	◯ < 2 years<br>◯ 2-5 years<br>◯ 5-10 years<br>◯ >10 years
Who alerts the intestinal rehabilitation team to consult on intestinal failure patients within the NICU?	☐ Primary NICU team (Neonatology)<br>☐ Dietician<br>☐ Surgery<br>☐ Gastroenterology<br>☐ Other
If other, who alerts the intestinal rehabilitation team to consult in your NICU?	__________________________________
Are you a part of the intestinal rehabilitation team?	◯ Yes<br>◯ No

Do you have criteria for consulting the intestinal rehabilitation team?	◯ Yes<br>◯ No
When does the intestinal rehabilitation team begin to follow the identified NICU patient?	☐ Upon bowel resection or ostomy formation<br>☐ Upon meeting definition of intestinal failure used by your institution<br>☐ Upon starting feeds<br>☐ Upon advancing feeds<br>☐ If unable to advance feeds<br>☐ When cholestasis develops<br>☐ When ready for transfer or discharge<br>☐ Other
If other, when does the intestinal rehabilitation team consult on patients?	__________________________________
How often does the intestinal rehabilitation team follow the identified neonate?	☐ Weekly<br>☐ Monthly<br>☐ As needed<br>☐ Close to discharge<br>☐ Other
If other, how often does the intestinal rehabilitation team see NICU intestinal failure patients?	__________________________________
Do you have a nutritional protocol (parenteral or enteral) or general care protocol for intestinal failure patients in the NICU?	◯ Yes<br>◯ No
What does your intestinal failure care protocol include? (check all that apply)	☐ Enteral nutrition<br>☐ Parenteral nutrition<br>☐ Lipid emulsion usage<br>☐ Laboratory monitoring<br>☐ Ethanol lock<br>☐ Other lock<br>☐ Other
What other topics/information does your intestinal failure nutritional protocol include?	__________________________________
What other lock is used other than ethanol for NICU intestinal failure patients?	__________________________________
Does your parenteral lipid emulsion usage include the following (check all that apply).	☐ Soybean lipid minimization<br>☐ Smoflipid<br>☐ Omegaven<br>☐ None of the above
Does your intestinal failure parenteral nutrition protocol include the following? (Check all that apply)	☐ GIR Maximum<br>☐ PN Cycling<br>☐ Limitation of trace mineral content<br>☐ Other
What other areas does your parenteral nutrition protocol include for intestinal failure in the NICU?	__________________________________

Does your enteral protocol include any of the following for intestinal failure patients in the NICU? (Check all that apply)	☐ Continuous feeds<br>☐ Specific formula or milk<br>☐ Rate of feeding advancement<br>☐ Threshold for ostomy or stool output<br>☐ Mucous fistula refeeding<br>☐ Enteral lipid usage<br>☐ Probiotics<br>☐ Other
What other topics does your enteral nutrition protocol include for intestinal failure patients in the NICU?	__________________________________
Where is home TPN teaching completed?	☐ in NICU<br>☐ on general GI or surgery floor<br>☐ we do not discharge patients home on TPN<br>☐ other
if other, where is home PN teaching completed?	__________________________________
If you do not discharge patients home on TPN, do you transfer patients to another facility?	◯ Yes<br>◯ No
If discharging home on PN, do you have a protocol for central venous access for home PN?	◯ Yes<br>◯ No
Who places central venous access for home PN? (check all that apply)	☐ Vascular access team<br>☐ Surgeons<br>☐ Interventional radiology<br>☐ Other
If other, who else places your central venous access for home PN?	__________________________________
Is there a preference for central venous access site for home PN?	☐ Internal jugular<br>☐ Upper limb<br>☐ Lower limb<br>☐ Any/no preference<br>☐ Other
If other, what is the other preferred site for home central venous access?	__________________________________
When discharging home, what mode(s) of home feeding are offered for neonatal intestinal failure patients? (check all that apply)	☐ Gastrostomy tube<br>☐ Nasogastric tube<br>☐ Jejunal feeds (via NJ or transpyloric tube)<br>☐ Gastrostomy-jejunostomy tube<br>☐ Continuous feeds<br>☐ PO<br>☐ Other
If other, what other enteral intake mode do you offer?	__________________________________
if discharging home on continuous feeds, do you offer continuous via NG?	◯ Yes<br>◯ No

Do you discharge/transfer neonates from the NICU with ostomies?	☐ Yes: Small intestine<br>☐ Yes: Large intestine (colostomy)<br>☐ No
What type of small intestine ostomy do you discharge home with? (check all that apply)	◯ Duodenostomy<br>◯ Jejunostomy<br>◯ Ileostomy<br>◯ Do not discharge home with small intestine ostomy only transfer
Where do you discharge/transfer to with an ostomy? (check all that apply)	☐ Transfer to another floor/unit in hospital<br>☐ Discharge home<br>☐ Another institution<br>☐ Other
If other, where else do you transfer or discharge to with an ostomy?	__________________________________
What are the criteria to consider discharge home with an ostomy?	__________________________________
If you do discharge home with an ostomy, where is the teaching completed? (check all that apply)	☐ NICU<br>☐ On general GI or surgery floor<br>☐ Other
if other, where is ostomy teaching completed?	__________________________________
Do you have a multidisciplinary intestinal failure rehabilitation clinic that follows these NICU patients outpatient?	◯ Yes<br>◯ No
If you do not have a multidisciplinary intestinal failure rehabilitation clinic, who follows these NICU intestinal failure patients outpatient?	☐ Gastroenterology<br>☐ Surgery<br>☐ Refer to another facility<br>☐ Depends on the patient<br>☐ Other
If other, who follows these NICU intestinal failure patients outpatient?	__________________________________
If yes, what specialties are included in the intestinal failure rehabilitation clinic? (check all that apply)	☐ Gastroenterology<br>☐ Surgery<br>☐ Pharmacy<br>☐ Dietitian<br>☐ Nurse<br>☐ Advanced Care Provider (NP/PA)<br>☐ Other
What other specialties help provide care in your intestinal failure rehabilitation clinic?	__________________________________

Are there additional specialties who offer care to patients in your intestinal failure rehabilitation outpatient clinic?<br>(Check all that apply)	☐ Speech therapy<br>☐ PT/OT<br>☐ Wound or ostomy nurse<br>☐ Interventional radiology<br>☐ Psychology<br>☐ Social Work<br>☐ Neurogastroenterology<br>☐ Hematology<br>☐ Nephrology<br>☐ Endocrinology<br>☐ Neonatology<br>☐ Other
If other, what additional specialities are offered in your outpatient intestinal failure rehabilitation clinic patients?	__________________________________
